# Preparation of a Novel Organic Phosphonic Acid Intercalated Phosphate Tailings Based Hydrotalcite and Its Application in Enhancing Fire Safety for Epoxy Resin

**DOI:** 10.3390/polym14040725

**Published:** 2022-02-14

**Authors:** Huali Zhang, Lingzi Jin, Hanjun Wu, Zhenyue Zhang, Junxia Yu, Wenjun Zhang, Yi Pan, Zhiquan Pan

**Affiliations:** 1 Engineering Research Center of Phosphorus Resources Development and Utilization of Ministry of Education, Key Laboratory for Green Chemical Process of Ministry of Education, School of Chemistry and Environmental Engineering, Wuhan Institute of Technology, Wuhan 430074, China; zhanghl413@126.com (H.Z.); jinlingzi1208@163.com (L.J.); zyzxmu@wit.edu.cn (Z.Z.); yujunxia_1979@163.com (J.Y.); fatchoy409@163.com (W.Z.); wuhj1204@cug.edu.cn (Y.P.); wuhj1204@126.com (Z.P.); 2 Hubei Provincial Engineering Research Center of Systematic Water Pollution Control, China University of Geosciences, Wuhan 430074, China; 3 School of Resources and Safety Engineering, Wuhan Institute of Technology, Wuhan 430074, China

**Keywords:** phosphate tailings, layered double hydroxides, epoxy resin, fire safety

## Abstract

Phosphate tailings (PTs) are solid waste, which is produced by phosphate flotation. In this work, PTs were used as raw materials for the preparation of diethylenetriamine pentamethronic acid (DTPMP) intercalated trimetal (Ca-Mg-Al) layered double hydroxides (TM-DTPMP LDHs) by co-precipitation method. TM-DTPMP LDHs were characterized by X-ray diffraction, fourier-transform infrared spectroscopy, scanning electron microscopy, differential thermal gravimetric analysis, X-ray photoelectron spectroscopy and applied as a flame retardant to improve the fire safety of epoxy resin (EP). The results showed that the composite materials exhibited obvious layered structure. After intercalation, layer spacing increased from 0.783 to 1.78 Å. When the amount of TM-DTPMP LDH in EP was 8%, the limitted oxygen index of the composite material increased from the original 19.2% to 30.2%. In addition, Cone calorimeter (CC) and Raman spectrum results indicated that with the addition of TM-DTPMP LDHs, the value of heat release rate peak (pHRR) and total heat release (THR) were reduced by more than 43% and 60%, while the value of smoke formation rate (pSPR) and the total smoke production (TSP) decreased nearly 64% and 83%, respectively. The significant reduction in the release of combustion heat and harmful smoke during EP combustion may be attributed to the synergistic flame-retardant effect between hydrotalcite and DTPMP. This work exhibited great potential for the green recycling of PTs and the enhancement of the fire safety of EP.

## 1. Introduction

As a typical and extensive phosphorus chemical solid waste, phosphorus tailings (PTs) are one of the main by-products produced in the process of phosphate mining and flotation. PTs are mainly composed of calcium and magnesium carbonates, and the main components are CaO, MgO and P_2_O_5_ [1,2]. According to incomplete statistics, 0.4 tons of PTs are produced for every ton of phosphate concentrate produced [3]. In China, nearly 9.5 million tons of phosphate tailings will be produced every year, but the comprehensive utilization rate of PTs is only about 7% [4]. A large amount of PTs can only be piled up in the tailings pond for a long time. After being washed by rainwater, this will lead to the chemical migration of harmful elements. As a result, it will cause serious pollution to the surrounding atmosphere, water and soil, destroy vegetation and even directly endanger the survival of humans and animals [5]. Therefore, the effective comprehensive utilization of PTs is imminent.

Epoxy resin (EP) is widely used in various industries, such as machinery, aerospace, adhesives, national defense, flooring and other industries due to its excellent chemical and physical properties. Therefore, the output of EP increases year by year. However, EP is a thermosetted thermoset resin based on its own chemical structure, the limit oxygen index in the air is only about 25.8%, and its application range is greatly limited. Thus, the flame retardancy modification of EP has always been an important research topic in academia and industry. In recent years, extensive studies have focused on the development of novel flame-retardant materials to improve the thermal stability of epoxy resins, such as halogen [6], phosphorus-nitrogen [7,8] and inorganic flame retardant (hydroxide) [9,10]. Among them, inorganic flame retardants are widely concerned because of their good smoke suppression effect and environmental protection [11,12,13].

Layered double hydroxides (LDHs) are regarded as a new type of eco-friendly and efficient flame retardant because of the formation of non-flammable gases H_2_O and CO_2_ and a metal oxide residue. More importantly, the introduction of organic anions or functional flame-retardant anions between the layers can effectively improve the flame-retardant efficiency of LDHs and its compatibility with polymer matrix materials. Zammarano et al. found that the flame resistance of LDH/EP nanocomposites is related to the level of dispersion as well as the intrinsic properties of the LDH derivative [14]. Ding et al. reported that the Cu-Al layered double hydroxide (Cu-Al-LDHs) and the sodium dodecyl sulfate (SDS) intercalated Cu-Al-LDHs (SDS-CuAl-(SDS)LDHs) can greatly improve the thermal stability and flame retardancy of EP [15]. Wang et al. found that functionalized LDHs based on multi-modifiers’ system composed by hydroxypropyl-sulfobutyl-beta-cyclodextrin sodium (sCD), dodecylbenzenesulfonate (DBS), taurine (T) and cardanol-BS showed excellent combustion safety and mechanical properties [16,17]. Jiang et al. found that Labyrinth effect of m-SiO_2_ and formation of graphitized carbon char catalyzed by Co-Al LDH played pivotal roles in the flame retardance enhancement [18]. Zhang et al. reported that a flame-retardant material composed of metal organic frameworks (MOFs) and LDHs can significantly improve its dispersion in EP and exhibit a good synergistic flame-retardant effect [19]. In addition, carbon nanotubes (CNTs) based organic nickel-iron layered double hydroxide (ONiFe-LDH-CNTs) can significantly improve the dispersion of CNTs and flame retardancy of LDHs in EP [20]. Meanwhile, as a typical organic phosphonic acid, Diethylenetriaminepenta (methylene-phosphonic acid) (DTPMP) has abundant C, N and phosphoric acid in its structure, which contribute to superior flame retardancy in high molecular polymers [21,22]. Therefore, DTPMP intercalated LDHs can not only effectively improve the compatibility between hydrotalcite and polymer substrate, but also improve the flame retardancy of the hydrotalcite. PTs contain a large amount of calcium and magnesium sources required for the synthesis of LDHs, which provides the possibility to prepare LDHs based on phosphate tailings.

Hence, this work introduced a favorable method to synthetise DTPMP intercalated trimetal layered double hydroxides (TM LDHs) used PTs by co-precipitation method. The effect of LDHs on the fire resistance of EP composites was evaluated by studying the thermal stability, flame retardancy, and smoke/CO release. The relevant mechanisms were provided by analyzing the gas and condensed phase of the EP matrix.

## 2. Materials and Methods

### 2.1. Materials

PTs was selected from a phosphorus chemical industry located in Jinmen Hubei province. MgCl_2_·6H_2_O, AlCl_3_·6H_2_O, NaOH, HCl (15%, *w*/*w*) and diaminodiphenylmethane (DDM) were all of analytical reagent grade quality and were purchased from Sinopharm Chemical Reagent Co., Ltd. (Shanghai, China) Diethylenetriamine pentamethronic acid (C_9_H_28_N_3_O_15_P_5_; DTPMP) was obtained from Aladdin Reagent Co., Ltd. (Shanghai, China). Epoxy resin (E-44, expoxy equiv 210–230 g/mol, hydrolysable chlorine ≤ 0.5%, inorganic chlorine ≤ 50 mg/kg, softening point 14–23 °C) was purchased by Nantong Xingchen Synthetic Material Co., Ltd. (Jiangsu, China). Deionized water was used in all experimentation.

### 2.2. Synthesis of TM-DTPMP LDHs/EP Composite Materials

The synthesis process included the following steps: (1) preparation of TM LDHs by co-precipitation method; (2) DTPMP intercalation of TM LDHs by ion-exchange method (TM-DTPMP LDHs); (3) preparation of TM-DTPMP LDHs/EP composites. The whole synthesis process was described below.

Preparation of TM LDHs from PTs. Generally, PTs were burned at 900 °C for three hours. Subsequently, 25 g of calcined PTs were dissolved in 90 mL HCl (15% *w*/*w*), stirred at 60 °C for 30 min, filtered, and then obtained acid solution. Subsequently, the NaOH solution (200 g/L) was used to adjust the pH of above acid solution to 5, then filtred to obtain pure solution. Then 20 mL pure solution was injected into 250 mL beakers, ensuring that the molar ratio of Ca^2+^ to Mg^2+^ was 1:2, Ca^2+^ and Mg^2+^ to Al^3+^ was 3:1, added 0.36 mol/L NaOH to the above pure solution for adjusting pH to 10 with strong stirring at 60 °C for 30 min. Then, the mixture was aged at 90 °C for 18 h, centrifuged, washed with ethyl alcohol absolute for three times, dryed at 55 °C vacuum oven for 48 h, and TM LDHs were obtained.

DTPMP intercalation of TM LDHs by ion-exchange method. An amount of 3 g of TM LDHs were soluted in 120 mL ultrapure water and then stir at 25 °C all day, which was named as solution A. Then sodium hydroxide solution was added into DTPMP (10 g) and adjusted pH to 5.5, stirred for 12 h at 25 °C, which was named as solution B. After that solution B was added dropwise to the solution A, stirred at 140 °C for 10 h. The precipitation was filtered and washed with ultrapure water until the supernatant became neutral, dried at 55 °C all day, and then TM-DTPMP LDHs were obtained.

Preparation of TM-DTPMP LDHs/EP composites. An amount of 2 g TM-DTPMP LDHs dispersed to 20 g EP, assisted with stirring, heated to 90 °C for 8 min, and following added 6 g DDM. After 10 min reaction, the EP based mixture solidified at 100 °C for 2 h and following solidified at 150 °C for 2 h. Lastly, TM-DTPMP/EP composites were obtained after cooling to room temperature.

### 2.3. Characterization

X-ray diffraction (XRD) was obtained (PANalytical, Almelo, The Netherlands) using Cu Kα ray with a scan speed of 2° (2θ) min^−1^. Scanning electron microscopy (SEM) images were investigated by SU 8010 (Hitachi, Tokyo, Japan) with an accelerating voltage of 20 Kv. The Fourier transform infrared spectroscopy (FT-IR) was performed using Nicolet iS50 (Thermo Scientific, Waltham, MA, USA) with the optical range of 400–4000 cm^−1^. The degree of graphitization of residual carbon was determined via a Laser confocal Raman Spectrometer (SPEX.1403). The thermogravimetric analysis (TGA) was measured using TA Q5000 (TA Co., Newcastle, DE, USA) at the heating rate of 10 °C min^−1^ under N_2_ condition with the flow rate of 40 mL min^−1^. An oxygen index tester (HC-2C, Nanjin, China) following GB/T2406.2-2009 standards detected the level of burning materials. The combustion heat release, effective heat, smoke generation and smoke toxicity were test by using Cone calorimeter (FTT0007, Fire Testing Technology, West Sussex, UK) followed iso 5660 standards and 35 KW radiation intensity. The sample sizes for cone calorimetry samples were cuboid 100 × 100 × 10 mm^3^.

## 3. Results and Discussion

### 3.1. Characterization of TM-DTPMP LDHs

#### 3.1.1. Chemical Properties of TM-DTPMP LDHs

**Figure 1:**
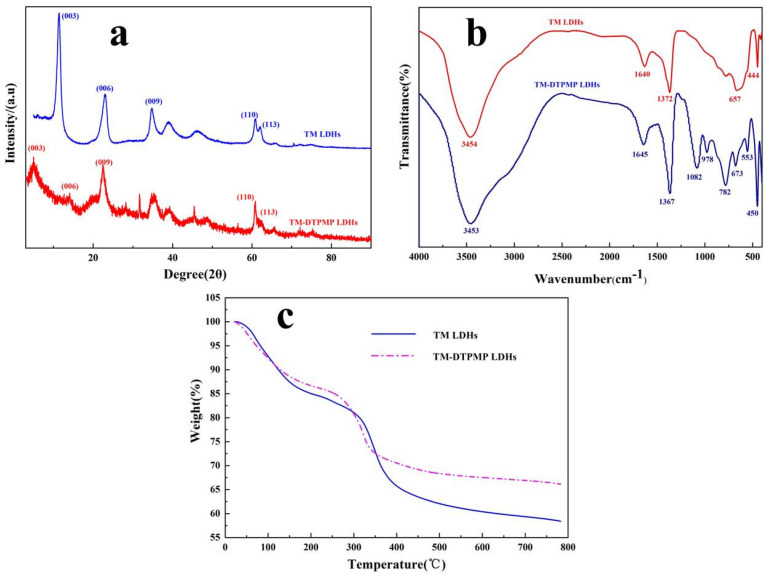
(**a**) XRD curves, (**b**) FT-IR spectras and (**c**) TG images of TM-DTPMP LDHs and TM LDHs.

The XRD of TM LDHs and TM-DTPMP LDHs composites were showed in Figure 1a. LDHs at (003), (006), (009), (110), and (113) represented the hydrotalcite structure [23]. The diffraction peaks of (003), (006) and (009) crystal planes can be seen at 11.3°, 22.97° and 34.83°, which conformed to the diffraction peak angles of TM LDHs XRD crystal planes [24,25]. The diffraction peaks of TM-DTPMP LDHs located at (003), (006), (009) were distinctly inclined to low angles. By Bragg’s law (2dsinθ = nλ), the basic distance d(003) of TM LDH and TM-DTPMP LDH was calculated to be 0.783 and 1.78Å, respectively [26]. The change of d(003) indicated that DTPMP anions may be inserted into the middle layer of TM LDHs.

As shown in Figure 1b, the FT-IR spectra of TM LDH and TM-DTPMP LDH have absorption peaks at 3454 cm^−1^, 3453 cm^−1^ and 1640, 1645 cm^−1^, which were attributed to the O-H band [27]. There were also strong characteristic peaks of CO_3_^2−^ in LDHs at 1367 cm^−1^ and 1372 cm^−1^ [28]. In the FT-IR spectrum of TM-DTPMP LDHs, the characteristic band at 782 cm^−1^ might be ascribed to the stretching vibration of C-P [29]. The presence of P-O and P=O stretching at 978 cm^−1^ and 1082 cm^−1^ indicated that DTPMP anions may be inserted into the TM-LDHs layer [30].

**Figure 2:**
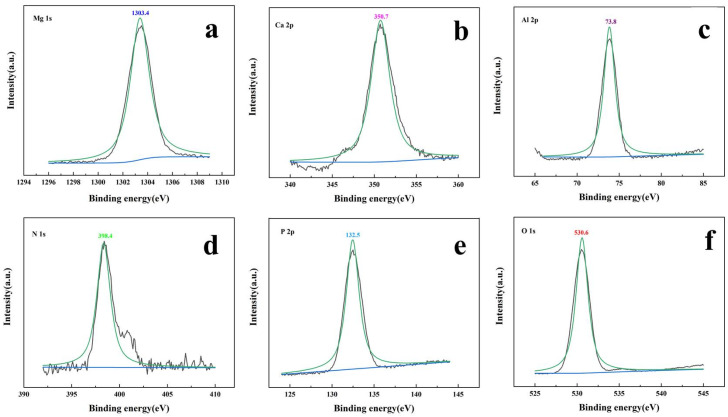
XPS survey for (**a**) Mg 1s, (**b**) Ca 2p, (**c**) Al 2p, (**d**) N 1s, (**e**) P 2p, and (**f**) O 1s.

XPS was used to analyze the chemical state of constituent elements and the change of valence. As shown in Figure 2, it was clear that the samples contained Mg, Al, Ca, O, N and P elements, which indicated that DTPMP might be inserted between the layers of hydrotalcite. The binding energies of Mg 1s were found at 1303.4 eV could be attributed to the active oxides of magnesium [31]. The binding energies of 350.7 eV and 73.8 eV could be explained by the existence of O-metal CaO and Al_2_O_3_, respectively [32,33]. In the N 1s XPS spectrum of TM-DTPMP LDH, there was only one peak at 398.4 eV, which was corresponded to C-N in DTPMP molecular [34]. The peak located at 132.8 eV assigned to P 2p could belong to the existence of P-O [35]. The peak located at 530.6 eV assigned to O 1s could be attributed to O^2−^ in CaO, MgO and Al_2_O_3_ (M-O represent the layered double hydroxides) [36].

#### 3.1.2. Microstructure of TM LDHs and TM-DTPMP LDHs

**Figure 3:**
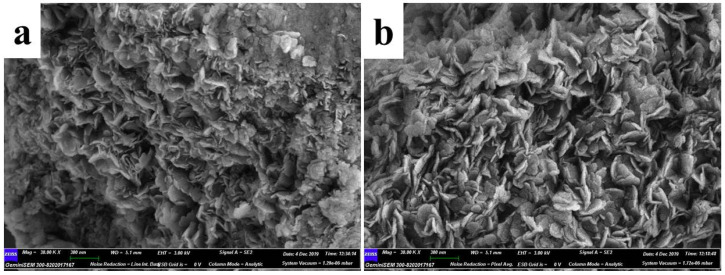
SEM images of (**a**) TM-DTPMP LDHs and (**b**) TM LDHs.

SEM image in Figure 3 showed the microstructure of the as-prepared TM LDHs and TM-DTPMP LDHs. It can be seen from Figure 3a,b that both TM LDHs and TM-DTPMP LDHs displayed obvious layered structure, preliminary hinted for the successful synthesis of hydrotalcite. TM LDHs exhibited layered structure of hydrotalcite-like with nuiform size and smooth surface. However, TM-DTPMP LDHs presented irregular layered structure with matte surface. The results indicated that the intercalation of DTPMP may damage the layered and crystal structure of LDHs.

#### 3.1.3. Thermal Stability of TM-DTPMP LDHs

The thermal stability of TM LDHs and TM-DTPMP LDHs were determined by TG. As shown in Figure 1c and Table 1, the weight loss of TM LDHs and TM-DTPMP LDHs below 200 °C could be explained by the removal of adsorbed water, which were attached to the surface of hydrotalcite due to the ambient humidity and incomplete drying [37]. During the crystallization process in the preparation of hydrotalcite, a large number of anions, such as carbonate, hydroxide and water molecules are inserted between the layers of LDHs. Meanwhile, the high degree of weight loss from 200–400 °C of TM LDHs and TM-DTPMP LDHs may be attributed to the removal of interlayer water and CO_3_^2−^ [38]. LDHs are mixed polymetal hydroxides. When hydrotalcite is calcined at a certain temperature and converted to a multi-metal mixed metal oxide, the hydroxide radicals in the structure will be converted into water molecules and overflow. Moreover, the initial temperature (T_5%_) and maximum weight loss temperature (T_max%_) of TM LDHs and TM-DTPMP LDHs were 102.3 °C, 328.4 °C and 103.5 °C, 325.1 °C, respectively. At the same time, the amount of char residual gradually increased from 60.7% of TM LDHs to 67.3% of TM-DTPMP LDHs. The results indicated that the intercalation of DTPMP contributed to the reduction of the decomposition temperature of composite materials. The structure of LDHs could gradually collapse with a temperature above 400 °C, which was followed by a loss of structure water and CO_3_^2−^ [39]. According to TG curves, the weight loss of TM LDHs at end point was higher than that of TM-DTPMP LDHs, which attributed to the incomplete thermal decomposition of DTPMP in TM-DTPMP LDHs and caused some carbon residues to remain in the layered double oxides (LDO). The results indirectly showed that a small amount of carbonate replaced by DTPMP, which was correlated with the chemical analysis.

### 3.2. Flame Retardant Performance Analysis of TM-DTPMP LDHs/EP Composites

#### 3.2.1. TG Analysis

**Figure 4:**
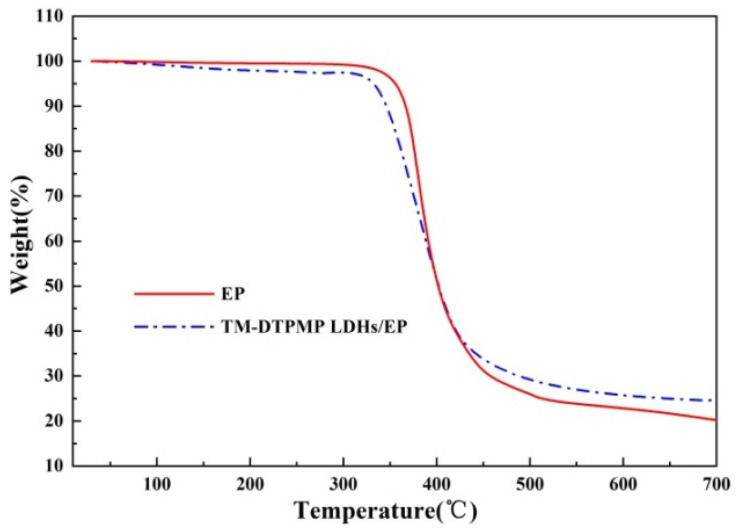
TG curves of EP and TM-DTPMP LDHs/EP.

The influence of TM-DTPMP LDHs/EP on the degradation behavior of EP was carried out by TG. It can be seen from Figure 4 and Table 1 that the initial decomposition temperature of EP composite decreases slightly with the addition of TM-DTPMP LDHs, which was mainly due to the high stability of EP matrix and deprivation of LDHs interlayer and adsorption H_2_O. The carbon production was an significant factors to judged the thermal stability of composites. When the addition quantity of TM-DTPMP LDHs was 8%, T_5%_ and T_max%_ of TM-DTPMP LDHs/EP samples increased to 340.3 and 385.6 °C, respectively. At the same time, the amount of char residual gradually increased from 22% of EP to 27.5% of TM-DTPMP LDHs/EP. A higher residue is beneficial for the flame retardant performance as the barrier is increased. In addition the flame retardant mechanism should start before the matrix decomposes, so that a decrease in decomposition temperature is expected.

#### 3.2.2. Analysis of Oxygen Index

The limited oxygen index (LOI) is one of the important indexes to evaluate the flame retardant properties of composite materials. Generally, the higher the value of LOI, the harder the composite materials is to burn. Figure S1 in Support Information (SI) displayed the change trend of the LOI under different TM-DTPMP LDHs additions. It showed that as the TM-DTPMP LDHs addition increased from 0% to 8%, the value of LOI increased from the initial value of 25.8% to 30.3%. The results indicated that TM-DTPMP LDHs was conducive to improving the flame retardant performance of EP. It was possible that the synergistic effect between TM LDH and DTPMP leads to a reduction in the total load of flame retardant additives, thereby improving the flame retardancy of the the composite materials [40]. Compared with other inorganic flame retardants in Table S1 [41,42,43], TM-DTPMP LDHs exhibited better flame retardant effect. Although the LOI value of the EP composites with only DTPMP was higher than that of the TM LDH and TM-DTPMP LDHs, the TM-DTPMP LDHs/EP composite still exhibited a better smoke suppression effect while maintaining a certain LOI value.

#### 3.2.3. Solid Phase Products of Composites after Combustion Analysis

**Figure 5:**
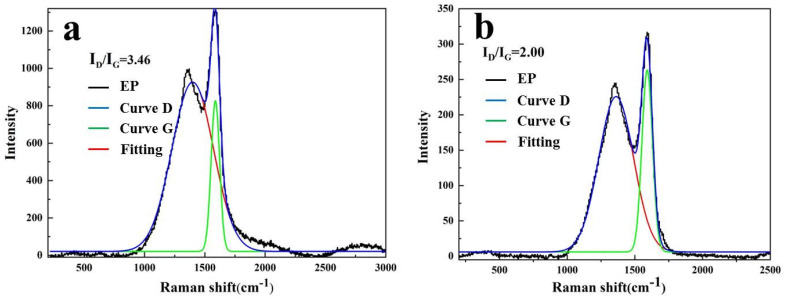
Raman spectra of residues of (**a**) EP and (**b**) TM-DTPMP LDHs/EP.

Raman spectrum was used to analyze the char yield and determine the carbonaceous quality of TM-DTPMP LDHs/EP. As shown in Figure 5, the peaks were observed at 1360 cm^−1^ and 1600 cm^−1^ may be caused by the vibration of carbon atoms from disordered graphite (D band) and graphitic carbon (G band), respectively. At the same time, the graphitization degree of composite materials was related to the area ratio of the D band and the G band (I_D_/I_G_) [44]. It can be seen from Figure 5a,b that the I_D_/I_G_ value (3.46) of pure EP was higher than that of TM-DTPMP LDHs/EP (2.00), which indicated that the EP matrix formed more residual carbon with a high graphitization degree under the addition of TM-DTPMP LDHs. Moreover, higher carbon yield may prevent the transport of degradation products to the combustion zone, thus reducing the heat release rate and inhibiting the transfer of heat to the degradation zone.

**Figure 6:**
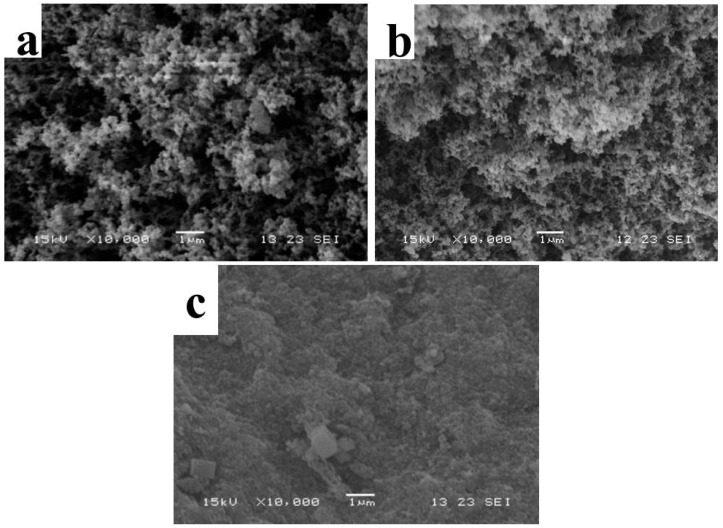
SEM images of (**a**) EP (**b**) TM-LDHs/EP and (**c**) TM-DTPMP LDHs/EP combustion products.

SEM images of the carbon residue reserved after combustion of TM-DTPMP/EP composites and pure EP were used to estimate the flame-retardancy mechanisms. As shown in Figure 6a–c, the carbon layer of pure EP was porous and disordered, while that of TM-DTPMP LDHs/EP was dense and flat. When adding LDHs alone, the surface of the EP after combustion showed a more compact carbon residue and metal oxides. As seen from EDS analysis of solid residue of TM-DTPMP LDHs/EP in Table S2, it was composed by Ca, Mg, Al, O, C, P and N. The dense and flat carbon layer acted as a barrier during the combustion process and can effectively inhibit the transfer of combustion heat and degradation products [45,46]. Moreover, the results showed that TM-DTPMPLDHs had obvious smoke suppression effect for EP, which was consistent with the results of cone calorimetry.

#### 3.2.4. Combustion Behavior Analysis of TM-DTPMP LDHs/EP Composites

**Figure 7:**
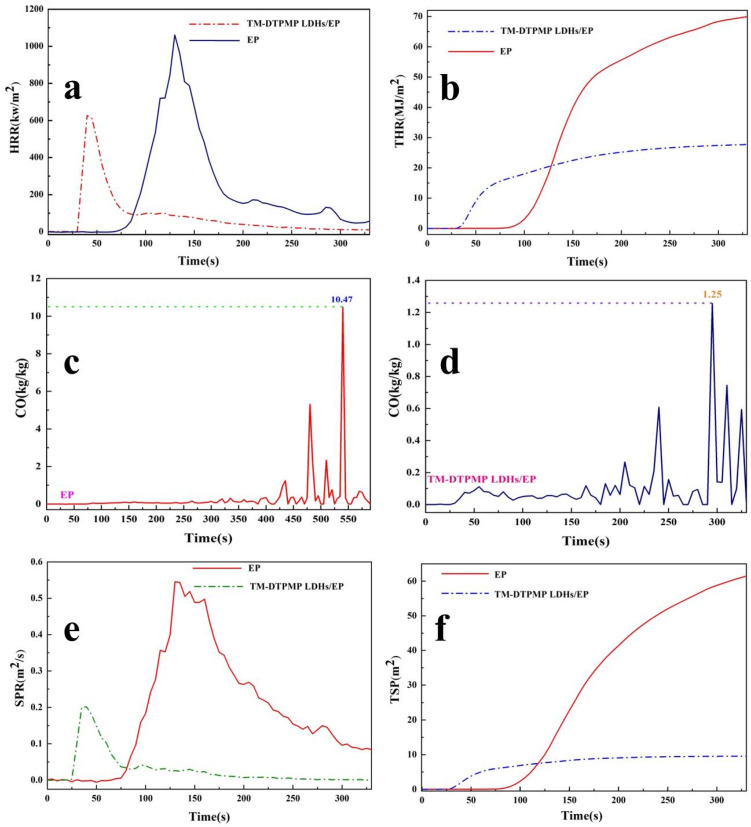
(**a**) HRR, (**b**) THR, (**e**) SPR, (**f**) TSP curves of EP and TM-DTPMP LDHs/EP; CO release rate curves of (**c**) EP and (**d**) TM-DTPMP LDHs/EP.

The heat release rate (HRR) and total heat release (THR) of EP and TM-DTPMP LDHs/EP obtained from cone calorimeter (CC) and were shown in Figure 7a,b. It can be seen that the heat release peak (pHRR) and THR curves of TM-DTPMP LDHs/EP decreased sharply, reducing by more than 43% and 60%, respectively. The decrease of pHRR indicated that TM-DTPMP LDHs could be effective in inhibiting the combustion of EP. In addition, the reduction of THR indicated that coke had been formed duriong the combustion of EP, which was related to the catalyze effect of TM LDHs and the synergistic effect between TM LDHs and the nitrogen/acid source in DTPMP [47]. Moreover, the addition of hydrotalcite reduced the ignition time but reduced the heat release, which may be attributed to the mixed oxide layer formed by the decomposition of the hydrotalcite and the decomposition of interlayer DTPMP [47].

CO release is an important factor in evaluating the safe combustion of EP, which discharged from partial combustion of oxygen-containing groups [48]. The CO release curves of EP and TM-DTPMP LDHs/EP were obtained from cone calorimeter (CC) and are shown in Figure 7c,d. It can be seen that the CO release rate of TM-DTPMP LDHs/EP was 1.25 kg/kg, far lower than that of pure EP (10.47 kg/kg), indicating that the addition of TM-DTPMP LDHs could be conducive to reducing the production of CO and enhancing combustion safety of EP. The results could be caused by TM LDHs catalyzed EP to reduce incomplete combustion of oxygen-containing groups.

Generally, dense smoke may suffocate humans and cause death, so smoke formation is one of the important factors in evaluating flame retardant performance [49]. The results of smoke formation rate (SPR) and the total smoke production (TSP) obtained from cone calorimeter (CC) were shown in Figure 7e,f. Compared with EP, the peak values of SPR (pSPR) and TSP reduced by nearly 64% and 83% for TM-DTPMP LDHs/EP, respectively. The obvious reduction of pSPR and TSR indicated that TM-DTPMP LDHs had a significant improvement on the combustion safety of EP.

#### 3.2.5. Flame Retardant Mechanism Analysis

**Figure 8:**
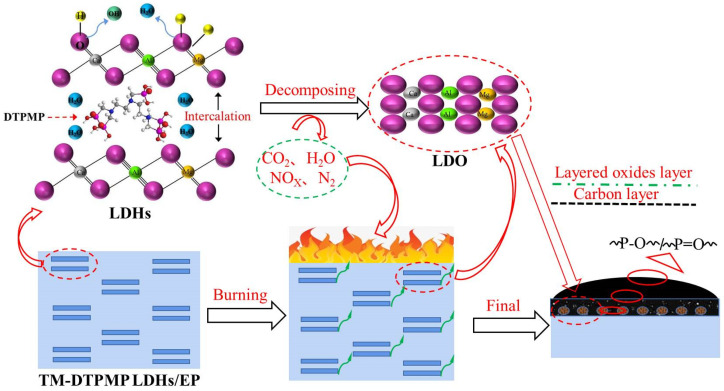
Possible flammability and charring process of TM-DTPMP LDHs/EP composites.

The mechanistic illustration of flame retardation of TM-DTPMP LDHs may be explained by Figure 8. The crystal water and CO_3_^2−^ between the layers of hydrotalcite, and the large amount of -OH on the laminates, make the hydrotalcite release CO_2_ and water when it was thermally decomposed, which can effectively dilute the concentration of combustible gas released and isolate oxygen [50]. The addition of TM-DTPMP LDHs promoted the decrease of smoke/CO and sped up the coke formation process, which may be attributed to the catalytic performance of LDHs. The metal hydroxides in TM LDHs converted to corresponding oxides (LDO) during combustion, especially Ca transforming to Ca-O, which can catalyze the decomposition of EP and promote the early cross-linking of the molecular chain. As a result, the carbonization process of EP and the smoke release during the combustion process were improved.

Moreover, P-N synergistic effect in the structure of DTPMP between the TM LDHs layers may also inhibit the combustion of EP. Phosphate will be pyrolyzed to produce pyrophosphoric acid, metaphosphoric acid and polymetaphosphoric acid, while nitride will be heated to release gaseous substances such as NH_3_, H_2_O and N_2_ [51]. Pyrophosphoric acid coats the substrate to form an isolation film. The non-combustible gas released by the compound and the isolation film will expand the substrate to form a foam layer, reducing the thermal conductivity. Meanwhile, phosphate also had the effect of catalyzing carbon formation, and the dense coking carbon structure had a blocking effect on combustion [52]. The synergistic effect of TM-LDH and DTPMP mainly occurs in the gas phase. DTPMP promotes the rapid decomposition of TM-LDH to release a large amount of water vapor and carbon dioxide, absorbs the heat of the substrate, reduces the surface temperature, and also dilutes the oxygen concentration and fiberboard heat. The effect of the concentration of the combustible gas produced by the solution makes the combustion proceed slowly and achieves the flame retardant effect. The gas phase flame retardancy of composite flame retardants is the result of the combined action of “cooling effect” and “dilution effect” [53]. Hence, the synergistic action between TM LDHs and DTPMP can effectively inhibit smoke and heat release and improve the fire safety of EP.

## 4. Conclusions

In this work, PTs were used as raw materials for preparation of TM-DTPMP LDHs by co-precipitation and ion exchange method, and further applied as flame retardant to enhance the fire safety of EP. The results indicated that the DTPMP intercalated hydrotalcite exhibited obvious layered structure and the interlayer spacing became larger. Compared with pure EP, the value of LOI increase from 19.2 to 30.2 with 8 wt% cotent of TM-DTPMP LDHs. The CC results indicated that the value of pHRR and THR decreased more than more than 43% and 60% with the addition of TM-DTPMP LDHs, respectively. Meanwhile, the values of pSPR and TSP reduced nearly 64% and 83%, which indicated the lower release of combustion heat and smoke during EP combustion. TM-DTPMP LDHs can effectively inhibit the release of smoke/CO and promote the formation of coke. The enhancement of TM-DTPMP LDHs to EP combustion safety may be mainly attributed to the conversion of LDHs to LDO, and the P-N synergistic effect in the structure of DTPMP between the TM LDHs layers. Therefore, TM-DTPMP LDHs prepared from PTs had great potential to be an eco-friendly and efficient flame retardant for improving the fire safety of EP.

## Figures and Tables

**Table 1:** Thermal decomposition parameters of TM LDHs, TM-DTPMP LDHs, EP and TM-DTPMP LDHs/EP.

Sample	T_5%_ (°C)	T_max_ (°C)	Residue at 750 °C (%)
TM LDHs	102.3	328.4	60.7
TM-DTPMP LDHs	103.5	325.1	67.3
EP	367.5	391.4	22
TM-DTPMP LDHs/EP	340.3	385.6	27.5

## Data Availability

The data presented in this study are available on request from the corresponding author.

## Supplementary Materials

The following supporting information can be downloaded at: https://www.mdpi.com/article/10.3390/polym14040725/s1, Figure S1: LOI trends curves of TM-DTPMP LDHs/EP, TM LDHs/EP and DTPMP/EP composites. Table S1: Different FR in Epoxy achieving the same LOI rating. Table S2: Elements content in different solid residue.

## References

[1] Wu J., Li J., Rao F. (2020). Mechanical property and structural evolution of alkali-activated slag-phosphate mine tailings mortars. Chemosphere.

[2] Nie Y., Dai J., Hou Y. (2020). An efficient and environmentally friendly process for the reduction of SO_2_ by using waste phosphate mine tailings as adsorbent. J. Hazard. Mater..

[3] Perumal P., Piekkari K., Sreenivasan H. (2019). One-part geopolymers from mining residues-Effect of thermal treatment on three different tailings. Miner. Eng..

[4] Chen Y., Wei Z., Irfan M. (2018). Laboratory investigation of the relationship between electrical resistivity and geotechnical properties of phosphate tailings. Measurement.

[5] Xiao Y., Xiang C., Lei H. (2019). Effect of change of Ca, P and Mg on the surface of catalyst prepared from phosphate tailing on urea alcoholysis. Catal. Commun..

[6] Lomakin S.M., Zaikov G.E. (1996). New type of ecologically safe flame retardant based on polymer char former. Polym. Degrad. Stabil..

[7] Chen L., Ruan C., Yang R. (2014). Phosphorus-containing thermotropic liquid crystalline polymers: A class of efficient polymeric flame retardants. Polym. Chem..

[8] Wang X., Hu Y., Song L., Xing W.Y., Lu H.D., Lv P., Jie G.X. (2010). Flame retardancy and thermal degradation mechanism of epoxy resin composites based on a DOPO substituted organophosphorus oligomer. Polymer.

[9] Hobbs C.E. (2019). Recent Advances in Bio-Based Flame Retardant Additives for Synthetic Polymeric Materials. Polymers.

[10] Sag J., Goedderz D., Kukla P., Greiner L., Schonberger F., Doring M. (2019). Phosphorus-Containing Flame Retardants from Biobased Chemicals and Their Application in Polyesters and Epoxy Resins. Molecules.

[11] Jiang W., Jin F.L., Park S.J. (2015). Synthesis of a novel phosphorus-nitrogen-containing intumescent flame retardant and its application to fabrics. J. Ind. Eng. Chem..

[12] Costes L., Laoutid F., Brohez S. (2017). Bio-based flame retardants: When nature meets fire protection. Mater. Sci. Eng. R.

[13] Chen M.J., Wang X., Tao M.C. (2018). Full substitution of petroleum-based polyols by phosphorus-containing soy-based polyols for fabricating highly flame-retardant polyisocyanurate foams. Polym. Degrad. Stabil..

[14] Zammarano M., Bellayer S., Gilman J.W., Franceschi M., Beyer F.L., Harris R.H., Meriani S. (2006). Delamination of organo-modified layered double hydroxides in polyamide 6 by melt processing. Polymer.

[15] Ding J.M., Zhang Y., Zhang X., Kong Q.H., Zhang J.H., Liu H., Zhang F. (2020). Improving the flame-retardant efficiency of layered double hydroxide with disodium phenylphosphate for epoxy resin. J. Therm. Anal. Calorim..

[16] Naderi Kalali E., Wang X., Wang D.Y. (2015). Functionalized layered double hydroxide-based epoxy nanocomposites with improved flame retardancy and mechanical properties. J. Mater. Chem. A.

[17] Wang X., Naderi Kalali E., Wang D.Y. (2015). Renewable Cardanol-Based Surfactant Modified Layered Double Hydroxide as a Flame Retardant for Epoxy Resin. ACS Sustain. Chem. Eng..

[18] Jiang S.D., Bai Z.M., Tang G., Song L., Stec A.A., Hull T.R., Hu Y. (2014). Synthesis of Mesoporous Silica@Co−Al Layered Double Hydroxide Spheres: Layer-by-Layer Method and Their Effects on the Flame Retardancy of Epoxy Resins. ACS Appl. Mater. Interfaces.

[19] Zhang Z.D., Qin J.Y., Yang R.J. (2020). Synthesis of a novel dual layered double hydroxide hybrid nanomaterial and its application in epoxy nanocomposites. Chem. Eng. J..

[20] Kong Q.H., Wu T., Tang Y.Q., Xiong L.M., Liu H., Zhang J.H., Guo R.H., Zhang F. (2017). Improving Thermal and Flame Retardant Properties of Epoxy Resin with Organic NiFe-Layered Double Hydroxide-Carbon Nanotubes Hybrids. Chin. J. Chem..

[21] Kiaei Z., Haghtalab A. (2014). Experimental study of using Ca-DTPMP nanoparticles in inhibition of CaCO_3_ scaling in a bulk water process. Desalination.

[22] Zhang H.L., Zhang J.X., Wu H.J., Pan Y., Xia Y., Pan Z.Q., Wang D.S. (2020). Synthesis and characterization of heterogeneous catalyst EDTMPA-Cu-LDH and study of the mechanism of visible-light photocatalytic degradation of Rhodamine B. Desalin. Water. Treat..

[23] Mantilla A., Jácome-Acatitla G., Morales-Mendoza G. (2011). Photoassisted Degradation of 4-Chlorophenol andp-Cresol Using MgAl Hydrotalcites. Ind. Eng. Chem. Res..

[24] Lestari F., Green A.R., Chattopadhyay G. (2006). An alternative method for fire smoke toxicity assessment using human lung cells. Fire Saf. J..

[25] Fang L., Li W., Chen H. (2015). Synergistic effect of humic and fulvic acids on Ni removal by the calcined Mg/Al layered double hydroxide. RSC Adv..

[26] Stojilovic N., Isaacs D.E. (2019). Inquiry-Based Experiment with Powder XRD and FeS_2_ Crystal: “Discovering” the (400) Peak. J. Chem. Educ..

[27] Zhang Y., Yang J., Fan F. (2019). Effect of Divalent Metals on the UV-Shielding Properties of M(II)/MgAl Layered Double Hydroxides. ACS Omega.

[28] Li J., Cui H., Song X. (2016). Adsorption and intercalation of organic pollutants and heavy metal ions into MgAl-LDHs nanosheets with high capacity. RSC Adv..

[29] Song J.X., Yu Z.X., Gordin M.L., Li X.L., Peng H.S., Wang D.H. (2015). Advanced Sodium Ion Battery Anode Constructed via Chemical Bonding between Phosphorus, Carbon Nanotube, and Cross-Linked Polymer Binder. ACS Nano.

[30] Fonder G., Minet I., Volcke C. (2011). Anchoring of alkylphosphonic derivatives molecules on copper oxide surfaces. Appl. Surf. Sci..

[31] Gao T., Hou S., Huynh K. (2018). Existence of Solid Electrolyte Interphase in Mg Batteries: Mg/S Chemistry as an Example. ACS Appl. Mater. Interfaces.

[32] Connell J.G., Genorio B., Lopes P.P. (2016). Tuning the Reversibility of Mg Anodes via Controlled Surface Passivation by H_2_O/Cl^−^ in Organic Electrolytes. Chem. Mater..

[33] Gao D.Q., Zhang J., Yang G.J., Zhang J.L., Shi Z.H., Qi J., Zhang Z.H., Xue D.S. (2010). Ferromagnetism in ZnO Nanoparticles Induced by Doping of a Nonmagnetic Element: Al. J. Chem. Phys..

[34] Mao N., Zhou C.H., Keeling J. (2018). Tracked changes of dolomite into Ca-Mg-Al layered double hydroxide. Appl. Clay Sci..

[35] Zhang W., He X., Ye G. (2014). Americium(III) capture using phosphonic acid-functionalized silicas with different mesoporous morphologies: Adsorption behavior study and mechanism investigation by EXAFS/XPS. Environ. Sci. Technol..

[36] Kim Y.J.P., Chong R. (2002). Analysis of Problematic Complexing Behavior of Ferric Chloride with N,N-Dimethylformamide Using Combined Techniques of FT-IR, XPS, and TGA/DTG. Inorg. Chem..

[37] Nayak S., Swain G., Parida K. (2019). Enhanced Photocatalytic Activities of RhB Degradation and H_2_ Evolution from in Situ Formation of the Electrostatic Heterostructure MoS_2_/NiFe LDH Nanocomposite through the Z-Scheme Mechanism via p-n Heterojunctions. ACS Appl. Mater. Interfaces.

[38] Wu H.J., Zhang H.L., Zhang W.J., Yang X.F., Zhou H., Pan Z.Q., Wang D.S. (2019). Preparation of magnetic polyimide@ Mg-Fe layered double hydroxides core-shell composite for effective removal of various organic contaminants from aqueous solution. Chemosphere.

[39] Wu H.J., Zhang W.J., Zhang H.L., Pan Y., Yang X.F., Pan Z.Q., Yu X.J., Wang D.S. (2020). Preparation of the novel g-C_3_N_4_ and porous polyimide supported hydrotalcite-like compounds materials for water organic contaminants removal. Colloid Surf. A.

[40] Liu Y., Gao Y.S., Zhang Z., Wang Q. (2021). Preparation of ammonium polyphosphate and dye co-intercalated LDH/polypropylene composites with enhanced flame retardant and UV resistance properties. Chemosphere.

[41] Guo X., Wang H.S., Ma D.L., He J.N., Lei Z.Q. (2018). Synthesis of a novel, multifunctional inorganic curing agent and its effect on the flame-retardant and mechanical properties of intrinsically flame retardant epoxy resin. Appl. Polym. Sci..

[42] Qian X.D., Song L., Yu B., Wang B.B., Yuan B.H., Shi Y.Q., Hu Y., Yuen R.K.K. (2013). Novel organic-inorganic flame retardants containing exfoliated graphene: Preparation and their performance on the flame retardancy of epoxy resins. J. Mater. Chem. A.

[43] Shi C.L., Qian X.D., Jing J.Y. (2021). Phosphorylated cellulose/Fe(3+)complex: A novel flame retardant for epoxy resins. Polym. Adv. Technol..

[44] Gupta S.S., Sreeprasad T.S., Maliyekkal S.M. (2012). Graphene from sugar and its application in water purification. ACS Appl. Mater. Interfaces.

[45] Zhou K., Wang B., Jiang S. (2013). Facile Preparation of Nickel Phosphide (Ni_12_P_5_) and Synergistic Effect with Intumescent Flame Retardants in Ethylene-Vinyl Acetate Copolymer. Ind. Eng. Chem. Res..

[46] Wang L., Yang W., Wang B. (2012). The Impact of Metal Oxides on the Combustion Behavior of Ethylene-Vinyl Acetate Coploymers Containing an Intumenscent Flame Retardant. Ind. Eng. Chem. Res..

[47] Lu H.D., Wilkie C.A. (2010). Study on intumescent flame retarded polystyrene composites with improved flame retardancy. Polym. Degrad. Stabil..

[48] Zhu Z.M., Lin P.L., Wang H., Wang L.X., Yu B., Yang F.H. (2020). A facile one-step synthesis of highly efficient melamine salt reactive flame retardant for epoxy resin. J. Mater. Sci..

[49] Zhu Z.M., Wang L.X., Lin X.B., Dong L.P. (2019). Synthesis of a novel phosphorus-nitrogen flame retardant and its application in epoxy resin. Polym. Degrad. Stabil..

[50] Mei Y.J., Xu J.X., Jiang L.H., Chen P., Tan Q.P. (2018). Protecting of steel from chloride-induced corrosion by cement slurry coatings with calcined Mg-Al layered double hydroxides. Mater. Rev..

[51] Zhang X., Li Y., Yan S. (2015). Improved flame-retardant properties of HIPS/ATH system by organo Fe-montmorillonite. Nanomater. Energy.

[52] Branca C., D’Angelo G., Crupi C. (2016). Role of the OH and NH vibrational groups in polysaccharide-nanocomposite interactions: A FTIR-ATR study on chitosan and chitosan/clay films. Polymer.

[53] Liang S.H., Zhang L.F., Chen Z.L., Fu F. (2017). Flame Retardant Efficiency of Melamine Pyrophosphate with Added Mg-Al-Layered Double Hydroxide in Medium Density Fiberboards. Bioresources.

