# Supplementary material for: Preparation of a Novel Organic Phosphonic Acid Intercalated Phosphate Tailings Based Hydrotalcite and Its Application in Enhancing Fire Safety for Epoxy Resin
Source: Polymers (Basel). 2022 Feb 14;14(4):725. doi: 10.3390/polym14040725 (PMC8875001; doi:10.3390/polym14040725)
Supplement: Supplementary file 1 [file polymers-14-00725-s001.zip › polymers-1552371-supplementary.pdf]

Article

Preparation of a Novel Organic Phosphonic Acid Intercalated Phosphate Tailings Based Hydrotalcite and Its Application in Enhancing Fire Safety for Epoxy Resin

Huali Zhang [1], Lingzi Jin [1], Hanjun Wu [1,2,*], Zhenyue Zhang [1,3], Junxia Yu [1], Wenjun Zhang [1], Yi Pan [1] and Zhiquan Pan [1]

[1] Engineering Research Center of Phosphorus Resources Development and Utilization of Ministry of Education, Key Laboratory for Green Chemical Process of Ministry of Education, School of Chemistry and Environmental Engineering, Wuhan Institute of Technology, Wuhan 430074, China; zhanghl413@126.com (H. Z.); jinlingzi1208@163.com (L.J.); wuhj1204@wit.edu.cn (H.W.); zyzxmu@wit.edu.cn (Z.Z.); yujunxia_1979@163.com (J.Y.); fatchoy409@163.com (W.Z.); wuhj1204@cug.edu.cn (Y.P.); whhj1204@126.com (Z. P.)

[2] Hubei Provincial Engineering Research Center of Systematic Water Pollution Control, China University of Geosciences, Wuhan 430074, China

[3] School of Resources and Safety Engineering, Wuhan Institute of Technology, Wuhan 430074, China

* Correspondence: wuhj1204@wit.edu.cn (H. W.)

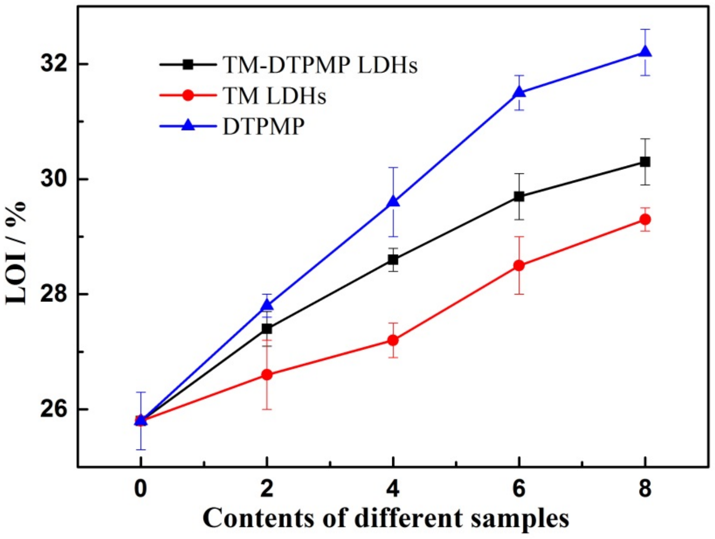

Figure S1. LOI trends curves of TM-DTPMP LDHs/EP, TM LDHs/EP and DTPMP /EP composites.

Table S1. Different FR in Epoxy achieving the same LOI rating

Sample	Adding amount (%)	LOI rating (%)	References
FCIN	9.0	31.0	(6)
FRs-rGO	5.0	29.5	(7)
Cell-P-Fe	10.0	29.1	(8)
TM LDH	9.2	30.1	this work
DTPMP	6.0	31.5	this work
TM-DTPMP LDHs	8.0	30.3	this work

Table S2. Elements content in different solid residue

Sample	O (%)	C (%)	N (%)	P (%)	Ca (%)	Mg (%)	Al (%)
TM LDHs/EP	26.22	59.17	-	-	5.18	5.81	3.62
DTPMP/EP	34.94	57.02	0.82	7.22	-	-	-
TM-DTPMP LDHs/EP	25.01	60.02	0.23	2.51	4.45	4.72	3.06
